# Dementia is a risk factor for major adverse cardiac and cerebrovascular events in elderly Korean patients initiating hemodialysis: a Korean national population-based study

**DOI:** 10.1186/s12882-017-0547-0

**Published:** 2017-04-06

**Authors:** Sung Min Jung, Clara Tammy Kim, Ea Wha Kang, Kyoung Hoon Kim, Shina Lee, Hyung Jung Oh, Seung-Jung Kim, Duk-Hee Kang, Kyu Bok Choi, Dong-Ryeol Ryu, Hyunwook Kim

**Affiliations:** 1 Department of Internal Medicine, College of Medicine, Tissue Injury Defense Research Center, Ewha Womans University, Seoul, Korea; 2 Graduate School of Public Health, Seoul National University, Seoul, Korea; 3 Department of Internal Medicine, NHIS Medical Center, Ilsan Hospital, Goyang-si, Gyunggi-do Korea; 4 Department of Public Health, Graduate School, Korea University, Seoul, Korea; 5 Department of Internal Medicine, Yonsei University College of Medicine Gangnam Severance Hospital, Seoul, Korea

**Keywords:** Dementia, Hemodialysis, Mortality, Ischemic stroke

## Abstract

**Background:**

Dementia is common in end-stage renal disease (ESRD) patients on hemodialysis (HD) and is associated with worse outcomes. This study aimed to investigate the risk of major adverse cardiac and cerebrovascular event (MACCE) in elderly patients with dementia initiating HD.

**Methods:**

Using the database from the Health Insurance Review & Assessment Service, we analyzed 10,171 patients aged 65 years or older who had initiated dialysis from 2005 to 2008. MACCE was defined as a composite outcome of all-cause mortality, nonfatal acute myocardial infarction, target vessel revascularization, and nonfatal ischemic and hemorrhagic stroke. The Kaplan-Meier method and Cox proportional hazards model were used, and further comparisons using propensity-score matching at 1:2 ratio were also performed.

**Results:**

A total of 303 elderly patients (3.0%) had dementia at initiating HD. During follow-up, dementia was a significant predictor of MACCE after adjustment for confounding variables. In addition, further analyzed in the propensity-score matched groups, dementia was an independent predictor of both nonfatal ischemic stroke and all-cause mortality.

**Conclusions:**

Dementia is an independent risk factor for mortality and ischemic stroke in elderly ESRD patients initiating HD. Patients with dementia who start dialysis should be closely monitored to reduce the risk of mortality and ischemic stroke.

**Electronic supplementary material:**

The online version of this article (doi:10.1186/s12882-017-0547-0) contains supplementary material, which is available to authorized users.

## Background

Dementia is a syndrome affecting memory, thinking and social abilities severely enough to interfere with normal activities of daily living [1]. It is the most common mental disorder in the elderly population and a major cause of death and disability among elderly individuals in the general population [2]. The World Health Organization reports that 47.5 million people have dementia, and there are 7.7 million new cases every year worldwide [1]. Recent studies revealed that cognitive impairment, including dementia, is more common in end-stage renal disease (ESRD) patients than in the general population [3–7]. This higher prevalence of dementia further deteriorates adverse outcomes of the ESRD patients, including the decline in functional status, hospitalization, dialysis withdrawal, and mortality [3, 8–10].

As populations are rapidly growing older, accompanied by the increasing prevalence of diabetes, hypertension, and cardiovascular diseases in most Western countries [11, 12], the requirement for dialysis treatment is increasing more rapidly in elderly patients than in younger ones [6, 13, 14]. Korea is also one of the most rapidly aging societies in the world. According to the 2014 annual report from the Korean Society of Nephrology – ESRD registry, the proportion of elderly patients (>65 years old) among those undergoing dialysis is markedly increasing, with the proportion rising up to 39.5% [15].

The decision whether and how to initiate dialysis is always not simple for any ESRD patient; for elderly patients with dementia, the decision is even more challenging. Many physicians hesitate to initiate dialysis in elderly patients with dementia because advanced age alone is the most important risk factor of death in ESRD patients [14, 16–18]; thus the benefits of dialysis would be further limited in elderly patients combined with dementia. These patients generally have difficulty to express their treatment-related symptoms. In addition, they are less likely to understand or to tolerate the dialysis process [8, 10, 19–21]. Several studies, including a recent nationwide population-based study in Taiwan, revealed that dementia is strongly and independently associated with morbidity and mortality not only in the general elderly population but also in ESRD patients [8, 10, 22–24].

Many studies have revealed that dementia and cerebrovascular disease are related [25–27]. Accumulating evidence from clinical, neuroimaging, and pathological studies showed a close link between dementia and cerebrovascular disease [25]. When examining patients who experienced a stroke, up to 10% of these patients already have dementia prior to the first stroke or would suffer from dementia during the first year after the onset of stroke [25, 28]. However, most studies exploring this relationship between dementia and cerebrovascular disease have been conducted exclusively in the general population.

A recent study revealed that the presence of cardiovascular disease is related to worse cognitive function in hemodialysis (HD) patients. However, this study only included the patients with a history of coronary disease or peripheral vascular disease but excluded those with a history of stroke [29]. In ESRD patients, the incidence rate of vascular dementia is similar to or rather exceed that of Alzheimer’s disease, which is in contrast with the general population [30, 31]. This supports the theory that atherosclerotic cerebrovascular disease plays a major role in the neuropathology of dementia in ESRD patients [3, 32]. However, there have been no studies exploring the contribution of dementia to a composite outcome consisting of mortality, cardiac events, and cerebrovascular diseases in ESRD patients receiving HD. Therefore, this study aimed to investigate the risk of major adverse cardiac and cerebrovascular events (MACCE) in elderly patients with dementia who initiate hemodialysis.

## Methods

### Data source and study population

The Korean Health Insurance Review and Assessment Service (HIRA) database was used. The details of the HIRA system were described elsewhere [33]. Data were collected from patients aged 65 years or older who started HD between January 1, 2005, and December 31, 2008. For incident dialysis patients, the early period, particularly within 90 days, after dialysis initiation is regarded as an unstable time because: 1) commonly, the patients were on temporary renal replacement therapy and the best dialysis modality is not yet determined; 2) adverse events during this period are frequently affected by various acute events, rather than by chronic intrinsic morbidities; 3) as a frequent target of comparison, the database for ESRD treatment provided by Medicare in the United States Renal Data System (USRDS) during that period was not readily available. Therefore, we adopted the 90-day rule suggested by USRDS [34] and only included the patients who had MACCE-free survival within the first 90 days after initiation of dialysis treatment. These patients were followed-up until December 31, 2009. The comorbid conditions of participants were identified by reviewing their medical record during a year before dialysis initiation. Mortality was confirmed by the Certificate Database (recorded data of reasons for changes in eligibility for National Health Insurance or Medical Aid, death, or emigration) and the National Health Insurance Claims Database. Other details of organization and use of Korean HIRA Service database were also provided elsewhere [33].

### Statistical analysis

Continuous variables and categorical variables were compared between elderly ESRD patients with and without dementia using the independent *t*-test and Chi-square test, respectively. The primary end-point of this study was the occurrence of MACCE during follow-up. MACCE was defined as a composite outcome of major adverse cardiac event (MACE) and nonfatal ischemic and hemorrhagic stroke [35]. MACE included all-cause mortality, nonfatal acute myocardial infarction, and target vessel revascularization, encompassing both percutaneous coronary intervention and coronary artery bypass graft. To assess event-free survival, the patients analyzed in this study were left-censored for the first event-free 90 days after dialysis initiation and were right-censored on December 31, 2009.

The incidence rates and 95% CIs were calculated using a Poisson distribution. The Kaplan-Meier method with log-rank tests was used to compare the event-free survival of MACCE among elderly ESRD patients with and without dementia. To identify risk factors of MACCE, Cox proportional hazards analysis was performed. The variables that have potential association with outcome were entered in the models, including dementia, age, sex, health insurance type, and comorbidities (diabetes mellitus, myocardial infarction, congestive heart failure, peripheral vascular disease, cerebrovascular disease, hemiparesis, chronic pulmonary obstructive disease (COPD), connective tissue disease, peptic ulcer disease, liver disease, and any cancer). Clinically relevant covariates and variables statistically significant in univariate analysis were considered as candidate variables in the multivariate Cox proportional hazard models. However, when the unadjusted and adjusted hazard ratios of dementia in elderly ESRD dialysis patients were estimated using the Cox proportional hazards model, there were significant differences in some baseline characteristics between dementia and no dementia groups. These differences may cause biased estimates of dementia effect on MACCE. Thus, 1:2 patient pairs with similar propensity scores were matched to reduce biases due to non-randomization, and further comparisons were then performed. All *P* values were 2-tailed, and *P* < 0.05 was considered significant. The statistical analyses were performed using statistic software SPSS, version 18.0 (SPSS Inc., Chicago, IL, USA) and R 3.1.2 (R Foundation for statistical computing) including the Matchit package.

## Results

### Baseline characteristics

This study included 10,171 patients who were 65 years or older, started HD between January 1, 2005, and December 31, 2008, and did not experience MACCE within 90 days from the date of dialysis initiation. Among them, 303 patients (3.0%) were identified as having an ICD-10 code of dementia. The prevalence of cerebrovascular disease and hemiparesis were higher in patients with dementia than in those without dementia (*P* < 0.001). The median follow-up duration was 37.8 months (range of 3–84 months).

The demographic characteristics between elderly ESRD patients with and without dementia are summarized in Table 1.

**Table 1:** Baseline characteristics of participants before and after propensity score matching

	Before matching			After matching		
	No Dementia (*n* = 9,868)	Dementia (*n* = 303)	*P*-value	No Dementia (*n* = 504)	Dementia (*n* = 252)	*P*-value
Age	71.7 ± 5.3	74.6 ± 5.9	<0.001	74.5 ± 5.6	74.6 ± 5.7	0.729
Male sex	5,346 (54.2)	136 (44.9)	0.002	243 (48.2)	111 (44.0)	0.315
Health security system						
National health insurance	8,840 (89.6)	261 (86.1)	0.067	446 (88.5)	214 (84.9)	0.203
Medical aid	1,028 (10.4)	42 (13.9)		58 (11.5)	384 (15.1)	
Comorbidities						
Diabetes	5,413 (54.9)	175 (57.8)	0.347	304 (60.3)	142 (56.3)	0.333
Cerebrovascular disease	1,521 (15.4)	131 (43.2)	<0.001	223 (44.2)	107 (42.5)	0.697
Hemiparesis	152 (1.5)	15 (5)	<0.001	29 (5.8)	12 (4.8)	0.691
Acute myocardial infarction	459 (4.7)	11 (3.6)	0.487	14 (2.8)	9 (3.6)	0.708
Congestive heart failure	1,829 (18.5)	70 (23.1)	0.053	115 (22.8)	60 (23.8)	0.831
Peripheral vascular disease	792 (8)	38 (12.5)	0.007	64 (12.7)	29 (11.5)	0.725
Chronic pulmonary disease	2,205 (22.3)	58 (19.1)	0.211	96 (19.5)	50 (19.8)	0.871
Connective tissue disease	314 (3.2)	7 (2.3)	0.491	9 (1.8)	4 (1.6)	1.000
Peptic ulcer disease	1,694 (17.2)	47 (15.5)	0.499	88 (17.5)	40 (15.9)	0.656
Liver disease	885 (9)	20 (6.6)	0.186	35 (6.9)	15 (6.0)	0.717
Any cancer	841 (8.5)	30 (9.9)	0.459	68 (13.5)	26 (10.3)	0.258

### Incidence Rates and Risk Factors of MACCE in All Elderly Patients Starting Dialysis

During the follow-up period, 54.2% of all elderly patients starting dialysis experienced MACCE, corresponding to an estimated incidence rate of 343 (95% CI, 333–352) events/1000 patient-years.

In multivariate Cox proportional hazards analysis for delineating independent risk factors associated with MACCE, dementia was statistically significant (HR, 1.258; 95% CI, 1.088–1.454; *P* = 0.002) after adjusting confounding variables in all elderly incident HD patients (Additional file 1: Table S1).

Age (HR, 1.033; 95% CI, 1.028–1.038; *P* < 0.001), male sex (HR, 1.106; 95% CI, 1.047–1.167; *P* < 0.001), medical aid (HR, 1.215; 95% CI, 1.119–1.319; *P* < 0.001), and comorbidities such as diabetes mellitus (HR, 1.201; 95% CI, 1.137–1.268; *P* < 0.001), cerebrovascular disease (HR, 1.473; 95% CI, 1.374–1.579; *P* < 0.001), hemiparesis (HR, 1.400; 95% CI, 1.164–1.684; *P* < 0.001), acute myocardial infarction (HR, 1.325; 95% CI, 1.180–1.488; *P* < 0.001), congestive heart failure (HR, 1.144; 95% CI, 1.070–1.222; *P* < 0.001), peripheral vascular disease (HR, 1.153; 95% CI, 1.049–1.268; *P* = 0.003), chronic pulmonary disease (HR, 1.067; 95% CI, 1.002–1.137; *P* = 0.045), and any cancer (HR, 1.472; 95% CI, 1.347–1.609; *P* < 0.001) were significant independent predictors of MACCE.

### Risks of MACCE and its components in all elderly HD patients with dementia

**Fig. 1:**
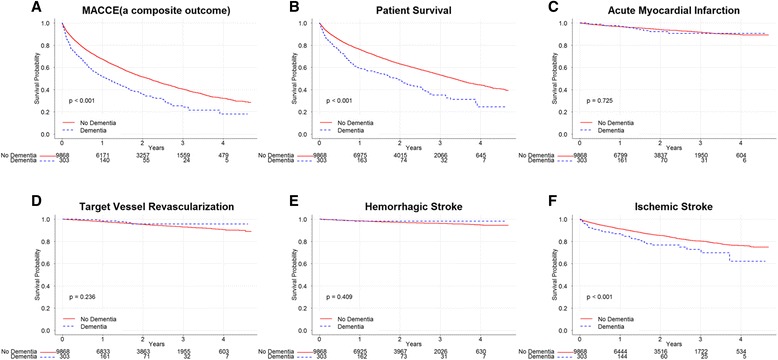
Kaplan–Meier event-free survival curves and comparisons between patients with or without dementia by log-rank test in all patients (*N* = 10,171). **a** The incidence of MACCE was significantly higher in patients with dementia than in those without dementia (*P* < 0.001). **b** Patients without dementia showed better survival rate than patients with dementia (*P* < 0.001). **c**-**e** There were no significant differences in event-free survival rates of nonfatal acute myocardial infarction, target vessel revascularization, or nonfatal hemorrhagic stroke (*P* = 0.725, *P* = 0.236, and *P* = 0.409, respectively). **f** However, incidence of nonfatal ischemic stroke was significantly higher in patients with dementia than in those without dementia (*P* < 0.001). MACCE, major adverse cardiac and cerebrovascular event

Kaplan-Meier survival curves according to dementia are shown in Fig. 1, which were compared by the long-rank test. The risk of MACCE was significantly higher in patients with dementia than in those without dementia (Fig. 1a, *P* < 0.001); the incidence rates of MACCE were 559 (95% CI, 485–644) events/1000 patient-years and 338 (95% CI, 328–347) events/1000 patient-years, respectively.

Among the MACCE endpoints, rates of all-cause mortality and ischemic stroke were significantly higher in patients with dementia compared to patients without dementia (Fig. 1, both *Ps* < 0.001), whereas rates of other MACCE endpoints were not significantly different between patients with and without dementia (Fig. 1).

A Cox proportional hazards regression model was performed to determine whether dementia is a significant predictor of composite outcome and separate components of MACCE in elderly HD patients (Table 2, before matching). Dementia was a risk factor of MACCE (HR, 1.258; 95% CI, 1.088–1.454; *P* = 0.002) and all-cause mortality (HR, 1.334; 95% CI, 1.140–1.562; *P* < 0.001). However, the statistical significance of nonfatal ischemic stroke in the univariate analysis did not persist in multivariate analysis (HR, 1.241; 95% CI, 0.931–1.654; *P* = 0.141). Dementia was not significant in univariate analysis for other individual MACCE endpoints, such as nonfatal acute myocardial infarction, target vessel revascularization, and nonfatal hemorrhagic stroke.

**Table 2:** Risks of major adverse cardiac and cerebrovascular events of participants before and after propensity score matching

	Before matching^a^			After matching^a^		
	HR	(95% CI)	*P*-value	HR	(95% CI)	*P*-value
MACCE	1.258	1.088–1.454	0.002	1.261	1.039–1.531	0.019
MACE	1.285	1.101–1.499	0.001	1.204	0.979–1.480	0.078
All-cause mortality	1.334	1.140–1.562	<0.001	1.301	1.053–1.607	0.015
Non-fatal acute myocardial infarction	0.994	0.581–1.701	0.982	0.589	0.264–1.314	0.196
Target vessel revascularization	0.612	0.272–1.378	0.236	0.424	0.144–1.246	0.119
Percutaneous intervention	0.525	0.216–1.275	0.154	0.319	0.094–1.077	0.066
Coronary artery bypass graft^b^	1.554	0.204–1.859	0.671	–	–	–
Non-fatal stroke	1.134	0.856–1.501	0.382	1.326	0.923–1.904	0.127
Non-fatal hemorrhagic stroke	0.551	0.204–1.492	0.241	0.556	0.154–2.010	0.371
Non-fatal ischemic stroke	1.241	0.931–1.654	0.141	1.492	1.027–2.168	0.036

*MACCE* major adverse cardiac and cerebrovascular event, *MACE* major adverse cardiac event, *HR* hazard ratio *CI* confidence interval

^a^Hazard ratio was adjusted for age, sex, insurance type, and comorbidities

^b^HR for coronary artery bypass graft was not calculated due to very low incidence

### Comparison of outcomes based on estimated propensity scores

To address concerns that the above results were affected by a huge heterogeneity in baseline comorbid conditions listed in Table 1 (before matching), propensity score matching was performed to adjust for all listed baseline differences between patients with and without dementia. After propensity score matching, the two groups were well matched for baseline characteristics (Table 1, after matching).

Kaplan-Meier survival curves (Additional file 2: Figure S1) and hazard ratios of baseline covariates for MACCE incidence (Additional file 1: Table S2) were estimated using the propensity score-matched group. Dementia was a significant predictor of MACCE (HR, 1.261; 95% CI, 1.039; *P* = 0.019) (Additional file 1: Table S2). In the analysis of individual MACCE end-points, the survival rate was significantly higher in patients without dementia than in those with dementia (Additional file 2: Figure S1B). The incidence of ischemic stroke was significantly lower in patients without dementia than those with dementia (Additional file 2: Figure S1F). In multivariate Cox proportional hazards model, dementia was an independent predictor of nonfatal ischemic stroke (HR, 1.492; 95% CI, 1.027–2.168; *P* = 0.036) and all-cause mortality (HR, 1.301; 95% CI, 1.053–1.607; *P* = 0.015) in propensity score-matched pairs (Table 2, after matching).

## Discussion

In this study, we investigated the risk of MACCE in elderly Korean patients initiating HD with dementia. Dementia was a significant predictor of MACCE. Among the individual endpoints of MACCE, dementia was an independent predictor of all-cause mortality and nonfatal ischemic stroke.

Several studies that examined the prevalence of cognitive impairment assessed by neuropsychological tests among ESRD patients indicate that the prevalence of cognitive impairment ranged from 16 to 38%, depending on characteristics of the included population [4, 5, 32, 36–40]. However, in studies that defined dementia by reviewing medical charts or billing codes, the prevalence of dementia was less than 15% [4, 36]. In our study, 3.2% of elderly patients starting dialysis were diagnosed with dementia based on a claims database. The much lower prevalence of dementia suggests possible under-diagnosis during the coding procedure [10]. It is well-known that stroke and cardiovascular risk factors significantly contribute to the development of dementia [25, 41]. A considerable number of studies in the past 30 years have shown the high prevalence of cognitive impairment after an onset of stroke in the general population [25]. Post-stroke dementia can be mediated by various mechanisms, including vascular cognitive impairment from neuroanatomical injuries in strategic brain regions and cerebral microbleeds [42]. However, many aspects of the effect of stroke on dementia are still obscure, and the effect of dementia on stroke also remains to be elucidated. Several studies suggest that dementia is more common in ESRD patients than in the general population [3–7], and the risk of stroke was also estimated to be five times higher in ESRD patients on dialysis compared to the general population [43]. Also, several studies revealed that the high morbidity and mortality was observed in ESRD patients with stroke and dementia [8, 10, 23, 24]. The relationship between stroke and dementia in renal patients has not been well clarified, but several studies recently indicated that vascular factors, particularly cerebrovascular disease, may play an important role the pathogenesis of dementia in ESRD [3, 32]. Interestingly, this close association between cognitive impairment and vascular diseases was also found in those with microalbuminuria as an early indicator of renal damage. This was demonstrated in the study by Vupputuri et al*.* in which cognitive dysfunction was only evident in the patients with an overt vascular disease but not in those without vascular disease [44]. The vascular structures of the brain and kidney showed similar anatomic and hemodynamic features in that both are end organs prone to vascular damage because of low-resistance and frequent exposure to a high blood flow volume [45]. Altogether, it can be deduced that dementia and ESRD share a common pathophysiologic process involving vascular injury in different end organs [32]. The high incidence rate of vascular dementia in elderly HD patients was also considered to be closely linked to advanced arteriosclerosis complicated by diabetes, hypertension, and rapid blood pressure changes during HD sessions [31]. Among other factors, atherosclerotic change and epithelium dysfunction were reported to have an important role in the process of cognitive impairment [22, 46]. Moreover, a recent study found that systemic atherosclerotic calcification, including the coronary calcification, was another factor associated with cognitive decline [47]. In this context, our finding that a high incidence of cerebrovascular disease in elderly ESRD patients with dementia can be, to some extent, explained (Table 1, before matching).

Other potential mechanisms, such as uremic toxin-induced neuronal injury, could also account for the development of dementia among ESRD patients. Of note, HD itself can be implicated in the pathogenesis of dementia *via* repeated exposures to rapid hemodynamic and metabolic changes during HD sessions, which leads to subclinical cerebral hypoperfusion, ischemia, and edema, and ultimately can potentiate cognitive decline [3]. This can explain why dementia is common among elderly people with HD and is getting worse as the duration of HD increases.

Our study has some limitations. As with other registry-based studies, there is a possibility of coding errors or misclassification of disease during processing of database, which can underestimate the incidence of endpoints. In addition, potential confounding factors for mortality such as critical laboratory findings, inflammatory or nutritional biomarkers, and dialysis doses were missing. However, the strength of our study lies in that it is based on a nationwide complete enumeration survey. We analyzed the cumulative outcomes of elderly ESRD patients with or without dementia from a large cohort that included nearly the entire population of dialysis patients with long follow-up periods (up to 84 months).

## Conclusion

Altogether, dementia is an independent risk factor of mortality and ischemic stroke in elderly ESRD patients initiating HD. Patients with dementia at the initiation of dialysis treatment should be closely monitored to reduce the risk of mortality and ischemic stroke.

## Additional files

Additional file 1: Table S1. Results of the Cox proportional hazards analysis for MACCE in all patients (*N* = 10,171). **Table S2.** Results of the Cox proportional hazards analysis for MACCE in propensity score-matched patients (*N* = 756). Abbreviations: MACCE, major adverse cardiac and cerebrovascular event. (DOCX 23 kb)

Additional file 2: Figure S1. Kaplan Kaplan–Meier event-free survival curves and comparisons between patients with and without dementia by log-rank test in propensity score-matched patients (*N* = 756). (A) The incidence of MACCE was significantly higher in patients with dementia than those without dementia (*P* = 0.0304). (B) Patients without dementia showed better survival rate compared to patients with dementia (*P* = 0.0348). (C-E) There were no significant differences in event-free survival rates of nonfatal acute myocardial infarction, target vessel revascularization, and nonfatal hemorrhagic stroke (*P* = 0.31, *P* = 0.133, and *P* = 0.402, respectively). (F) However, the incidence of nonfatal ischemic stroke was significantly higher in patients with dementia than those without dementia (*P* = 0.0492). Abbreviations: MACCE, major adverse cardiac and cerebrovascular event. (TIF 476 kb)

## Abbreviations

COPD: Chronic pulmonary obstructive disease
ESRD: End-stage renal disease
HD: Hemodialysis
HIRA: The Korean Health Insurance Review and Assessment Service
ICD: International Classification of Diseases
MACCE: Major adverse cardiac and cerebrovascular event
MACE: Major adverse cardiac event
NRF: The National Research Foundation of Korea
