# Supplementary material for: Dementia is a risk factor for major adverse cardiac and cerebrovascular events in elderly Korean patients initiating hemodialysis: a Korean national population-based study
Source: BMC Nephrol. 2017 Apr 6;18:128. doi: 10.1186/s12882-017-0547-0 (PMC5382664; doi:10.1186/s12882-017-0547-0)
Supplement: Supplementary file 1 — Results of the Cox proportional hazards analysis for MACCE in all patients (N = 10,171). Table S2. Results of the Cox proportional hazards analysis for MACCE in propensity score-matched patients (N = 756). Abbreviations: MACCE, major adverse cardiac and cerebrovascular event. (DOCX 23 kb) [file 12882_2017_547_MOESM1_ESM.docx]

**Table S1. Results of the Cox proportional hazards analysis for MACCE in all patients (N=10,171).**

|  | Univariate analysis | | | Multivariate analysis* | | |
| --- | --- | --- | --- | --- | --- | --- |
|  | HR | (95% CI) | *P*-value | HR | (95% CI) | *P*-value |
| Dementia | 1.588 | 1.377 – 1.832 | < 0.001 | 1.258 | 1.088 – 1.454 | 0.002 |
| Age (per 1-yr increase) | 1.033 | 1.028 – 1.038 | < 0.001 | 1.033 | 1.028 – 1.038 | < 0.001 |
| Male (vs. female) | 1.109 | 1.052 – 1.170 | < 0.001 | 1.106 | 1.047 – 1.167 | < 0.001 |
| Medical aid (vs. National health insurance) | 1.231 | 1.134 – 1.335 | < 0.001 | 1.215 | 1.119 – 1.319 | < 0.001 |
| Diabetes mellitus | 1.157 | 1.097 – 1.221 | < 0.001 | 1.201 | 1.137 – 1.268 | < 0.001 |
| Cerebrovascular disease | 1.607 | 1.504 – 1.717 | < 0.001 | 1.473 | 1.374 – 1.579 | < 0.001 |
| Hemiparesis | 1.786 | 1.493 – 2.137 | < 0.001 | 1.400 | 1.164 – 1.684 | < 0.001 |
| Acute myocardial infarction | 1.459 | 1.300 – 1.636 | < 0.001 | 1.325 | 1.180 – 1.488 | < 0.001 |
| Congestive heart failure | 1.210 | 1.133 – 1.292 | < 0.001 | 1.144 | 1.070 – 1.222 | < 0.001 |
| Peripheral vascular disease | 1.230 | 1.119 – 1.351 | < 0.001 | 1.153 | 1.049 – 1.268 | 0.003 |
| Chronic pulmonary disease | 1.122 | 1.053 – 1.195 | < 0.001 | 1.067 | 1.002 – 1.137 | 0.045 |
| Connective tissue disease | 1.068 | 0.920 – 1.238 | 0.388 | – | – |  |
| Peptic ulcer disease | 1.040 | 0.970 – 1.116 | 0.270 | – | – |  |
| Liver disease | 1.040 | 0.947 – 1.141 | 0.414 | – | – |  |
| Any cancer | 1.480 | 1.355 – 1.617 | < 0.001 | 1.472 | 1.347 – 1.609 | < 0.001 |

* HR was adjusted for all parameters with < 0.10 of *P*-value in the univariate analysis.

MACCE, major adverse cardiac and cerebrovascular event; HR, hazard ratio; CI, confidence interval.

**Table S2. Results of the Cox proportional hazards analysis for MACCE in propensity score-matched patients (N=756).**

|  | Univariate analysis | | | Multivariate analysis* | | |
| --- | --- | --- | --- | --- | --- | --- |
|  | HR | (95% CI) | *P*-value | HR | (95% CI) | *P*-value |
| Dementia | 1.236 | 1.020 – 1.499 | 0.031 | 1.261 | 1.039 – 1.531 | 0.019 |
| Age (per 1-yr increase) | 1.027 | 1.012 – 1.043 | 0.000 | 1.033 | 1.017 – 1.049 | 0.000 |
| Male (vs. female) | 1.145 | 0.954 – 1.373 | 0.146 | 1.050 | 1.868 – 1.270 | 0.616 |
| Medical aid (vs. National health insurance) | 1.041 | 0.794 – 1.365 | 0.771 | 1.041 | 0.787 – 1.376 | 0.779 |
| Diabetes mellitus | 0.955 | 0.794 – 1.149 | 0.626 | 0.998 | 0.826 – 1.205 | 0.980 |
| Cerebrovascular disease | 1.446 | 1.205 – 1.736 | 0.000 | 1.411 | 1.166 – 1.706 | 0.000 |
| Hemiparesis | 1.641 | 1.140 – 2.364 | 0.008 | 1.377 | 0.939 – 2.021 | 0.102 |
| Acute myocardial infarction | 1.267 | 0.757 – 2.120 | 0.368 | 1.080 | 0.640 – 1.822 | 0.773 |
| Congestive heart failure | 1.155 | 0.936 – 1.424 | 0.178 | 1.094 | 0.884 – 1.354 | 0.407 |
| Peripheral vascular disease | 1.746 | 1.348 – 2.262 | 0.000 | 1.733 | 1.331 – 2.257 | 0.000 |
| Chronic pulmonary disease | 1.319 | 1.054 – 1.651 | 0.015 | 1.298 | 1.035 – 1.627 | 0.024 |
| Connective tissue disease | 1.543 | 0.797 – 2.988 | 0.198 |  | – |  |
| Peptic ulcer disease | 0.941 | 0.736 – 1.203 | 0.627 |  | – |  |
| Liver disease | 1.216 | 0.861 – 1.716 | 0.267 |  | – |  |
| Any cancer | 1.350 | 1.038 – 1.755 | 0.025 | 1.349 | 1.033 – 1.762 | 0.028 |

* HR was adjusted for all parameters with < 0.10 of *P*-value in the univariate analysis.

MACCE, major adverse cardiac and cerebrovascular event; HR, hazard ratio; CI, confidence interval.
