# Supplementary figures and images for: Dementia is a risk factor for major adverse cardiac and cerebrovascular events in elderly Korean patients initiating hemodialysis: a Korean national population-based study
Source: BMC Nephrol. 2017 Apr 6;18:128. doi: 10.1186/s12882-017-0547-0 (PMC5382664; doi:10.1186/s12882-017-0547-0)

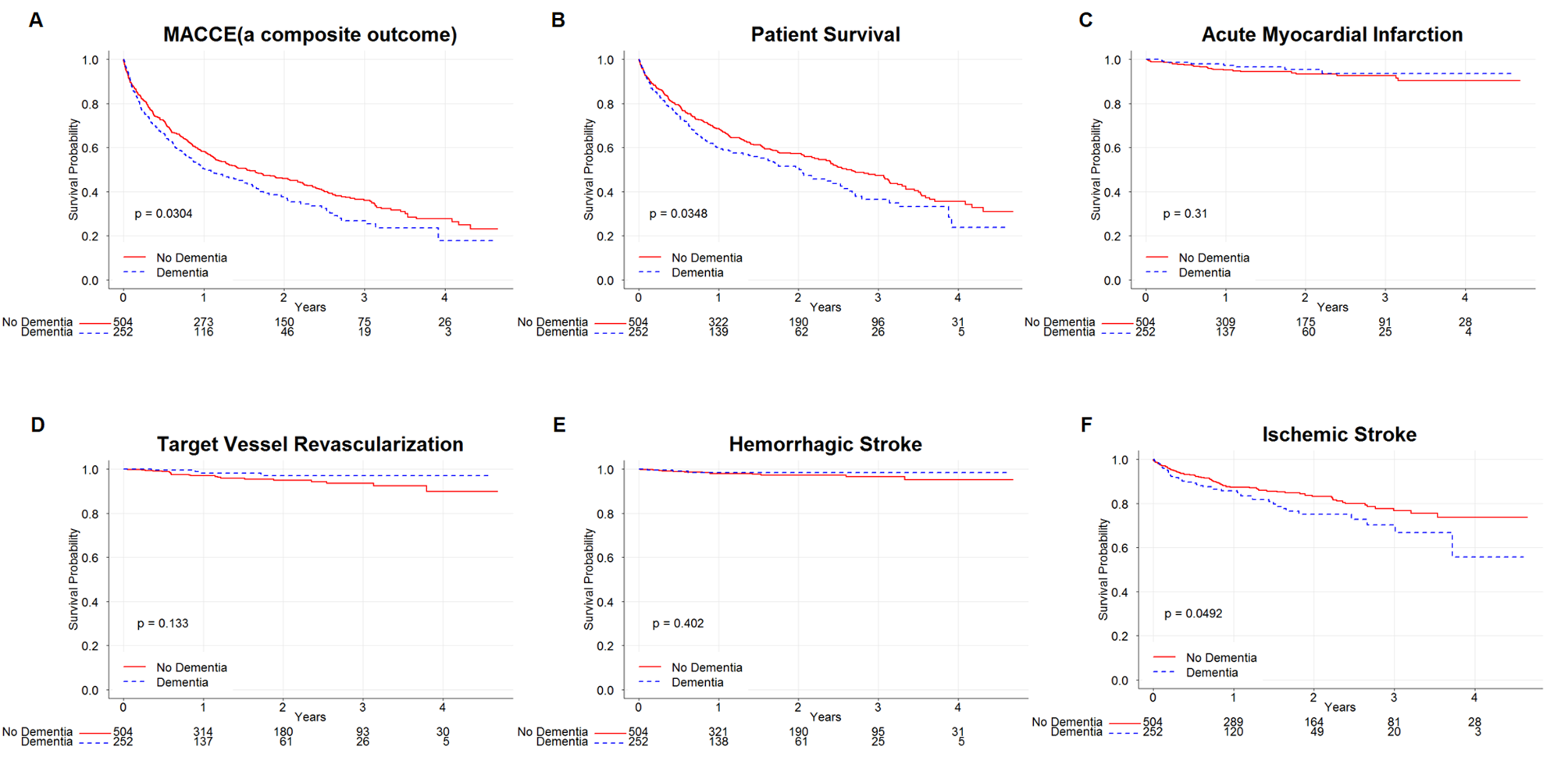

Supplement: Supplementary file 2 — Kaplan Kaplan–Meier event-free survival curves and comparisons between patients with and without dementia by log-rank test in propensity score-matched patients (N = 756). (A) The incidence of MACCE was significantly higher in patients with dementia than those without dementia (P = 0.0304). (B) Patients without dementia showed better survival rate compared to patients with dementia (P = 0.0348). (C-E) There were no significant differences in event-free survival rates of nonfatal acute myocardial infarction, target vessel revascularization, and nonfatal hemorrhagic stroke (P = 0.31, P = 0.133, and P = 0.402, respectively). (F) However, the incidence of nonfatal ischemic stroke was significantly higher in patients with dementia than those without dementia (P = 0.0492). Abbreviations: MACCE, major adverse cardiac and cerebrovascular event. (TIF 476 kb) [file 12882_2017_547_MOESM2_ESM.tif]
